# The Approach of Cholera

**Published:** 1891-01-17

**Authors:** 


### The Practitioner's Mirror and Retrospect.

*[The Editor will be glad to receive offers of co-operation and contributions from members of the profession. There is no doubt that much valuable material full of interest and instruction is at present lost to science. This is largely so in the case of (1) the younger and less-known members of the hospital staff (2) those connected with cottage hospitals, and (3) the great body of medical practitioners not attached to any institution. The co-operation of all is cordially invited to enable the Editor to make* The Hospital *of the utmost possible use and value to the profession. Specialists willing to review books on their special subjects are invited to communicate this fact at once. All letters should be addressed to* The Editor, The Lodge, Porchester Square, London, W.]

### MEDICINE.

### THE APPROACH OF CHOLERA.

As during recent years has so often occurred, we may soon again have news of cholera on the Continent. People will be saying that it is introduced from the East, and the Government of India will be blamed for not preventing the spread of the disease from where some people call its "home" in Hindustan to Europe. But there is no proof whatever that cholera has ever spread from India to Europe. The idea that cholera originates in the East, and spreads from East to West, has arisen, because after an epidemic in India it was discovered that cases had occurred in Afghanistan, in Turkestan, or in other countries, more or less on the route to Europe. But if the countries to the west of India had been similarly re-searched, there would have been quite as much evidence of the spread of the disease in a westerly direction. This, however, was not practicable. The countries to the west of India were comparatively little known, and could not be searched for evidences, like the countries to the East. We cannot accept the dictum that because a case of cholera occurs in Afghanistan, or in any other country this year, it is the onward march of last year's Indian cholera. As a matter of experience and observation there is more reason for the belief that cholera occurs at certain seasons in all countries, although not ordinarily in the same degree of intensity. Some time ago, towards the end of last year, Sir William Moore wrote an article (*British Medical Journal*) "On the Analogy of Summer Diarrhœa and Cholera." Sir W. Moore believes summer diarrhœa and cholera to be the same disease. He says many cases of cholera commence as diarrhœa. That terming a malady cholera only when certain symptoms occur is not consistent with the variations which we recognise in the symptoms of many other diseases. That cholera is in its manifestations protean. That many cases of diarrhœa occurring at times when there is no cholera, would be unhesitatingly returned as cholera if the latter malady prevailed. That the pathology of cholera and summer diarrhœa is identical. That both prevail in hot seasons. That Asiatic cholera is not, as supposed by many, a disease unknown previous to 1817, but that it has prevailed both in Europe and Asia from the earliest periods. "Experience has taught me not to expect to be able to recognize Asiatic malignant, or true cholera as it is distinctively termed, as a disease always characterised by rice-water evacuations, cramps, cyanosis, and other symptoms or phenomena attributed to it in text-books; but to prepare to meet it in insiduous forms, especially in that of diarrhœa. Mere degree is not sufficient for considering a disease different in essential character. . . . The question at what stage do sufferers from diarrhœa become the victims of cholera has never been satisfactorily answered." Sir W. Moore might also have added that there are other questions connected with cholera which have never been satisfactorily answered. For instance, the question whether or not it is contagious has never been settled; numbers of instances might be cited when contagion appears to have been the means of communication; meaning by contagion actual contact with the sick. On the other hand there is quite as forcible evidence forthcoming against this theory. Especially that found in the fact that attendants on cholera patients do not suffer more from the malady than other people. Again, whether or not it is infectious has not been decided—meaning by infection, communication through the atmosphere without actual contact. When, some years back, Koch announced the discovery of a cholera bacillus, not a few accepted the bacillus as the cause of cholera. There is, however, a growing belief that the bacillus has nothing to do with the causation of the disease; for it has been found when and where cholera was not. All that we can possibly say with reference to the causation of cholera is that some peculiar atmospheric condition is a necessity for its development, just as a peculiar atmospheric condition appears a necessity for the development of the kindred malady, summer diarrhœa. Also that some peculiar idiosyncracy, temperament, or constitution, is more susceptible to the influence of such atmospheric conditions than are others. There are theories as to what the peculiar atmospheric condition is, and also as to the constitution most liable to attack, but nothing is known for certain, and it appears more desirable to confess our ignorance than to endeavour to cloak it in scientific jargon and theory. By expression of ignorance we at least leave the road open to future reception of facts. We give one instance of theorising on cholera. Dr. Patrick Behir publishes an article on the Etiology of Cholera in a recent number of the *Indian Medical Gazette.* The susceptibility or invulnerability to the effects of the cholera poison he believes to be largely due to the state of reaction of the digestive juices at the time the *materies morbi* is brought into contact with them. The greater the alkalinity the greater the liability to the disease. Nineteen experiments on dogs were made. The dogs were fed on cholera excrement mixed with their food, 20 grains each of carbonate of magnesia and bicarbonate of potash being added. In three of the experiments there was produced a disease similar to cholera. Dr. Behir regards the practical outcome of the experiments to be, that so long as we keep up a certain degree of acidity in the gastric juice, we impart an immunity to the action of the poison. He therefore advocates the use of disinfectant acid as a prophylactic measure when cholera prevails. The plan followed has been to administer drachm doses of sulphurous acid. The efficacy of the dilute sulphuric acid and opium in the treatment of incipient cholera, and in that form of diarrhœa which prevails so extensively when cholera is about, may be explained on the same hypothesis. Now treating diarrhœas and choleras by sulphuric and sulphurous acid is by no means a new practice. It has often been spoken of favourably, and it has been as frequently condemned. Unfortunately, the remedies which seem to act beneficially during one epidemic of cholera often prove useless in the next. An eminent Indian medical officer, the late Dr. Norman Chevers, who probably had as great an experience of cholera as any man, stated that the last epidemic of the disease he witnessed was not only different in various characteristics to what he had ever met with before, but that no treatment satisfactory in other epidemics proved beneficial in the last. To give an account, or even a list, of the various treatments which have been proposed would be waste of time. From copious bleeding to antimony and purgatives, from calomel to cold water, all have been tried and found wanting. Still, we find medical men extolling some particular line of treatment, or some special drug. Here is an example: In the same journal (*Indian Medical Gazette*) we notice an article on the treatment of cholera by oil of eucalyptus, by Mr. E. Harold Brown, of the Bengal Medical Service. The dose of the oil for an adult was five minims in a little milk, that for children half a minim upwards, given every quarter of an hour for the first hour, and afterwards hourly. In the majority of cases the first effect was early cessation of vomiting, purging being restrained some time later. The author believes the good results depend on the stimulant and antiseptic properties of the oil. The mortality of the total cases treated was 19·04 per cent., and the author states the epidemic was not a slight one, there having been 126 cases. In an adjoining locality, out of 117 cases occurring and treated differently, the death ratio was 56·41 per cent. And so on, numbers of instances might be brought forward tending to show the good results of some particular treatment, which proves of little value when used again.

Can nothing, therefore, be done in a case of cholera? To which we reply that a great deal may be done, and that cholera patients are often saved. But they are not saved by drugging. First, when cholera is prevailing, the slightest symptom of diarrhœa should be attended to. It does not much matter what is given, provided the diarrhœa is treated in a rational manner. Chevers preferred tannic acid (ten grains after the first motion, and five grains after every other). Playfair advised twenty drops of laudanum and three grains of cayenne pepper in half a tumbler of hot water. Macnamara used one grain of opium with four of acetate of lead. Moore recommends thirty drops of chlorodyne in a half wine glass of brandy with as much water, which are remedies usually to be found when wanted. If purging continues ten grains of Dover's powder every three hours. Some authorities recommend also diuretics, with the view of maintaining the flow of urine. If vomiting occurs, a mustard poultice should be applied to the epigastrum, and recollecting that the incidence of cholera is chiefly on the lower part of the small intestines the right iliac fossa should be counter-irritated. But in any case the patient should be kept *from the first in the recumbent posture.* And until purging has stopped all solid food should be avoided. Iced water may be allowed in moderation; cramps are best relieved by friction. If collapse occurs, the period for any medicine has now passed away. But as Surgeon-General Murray says, every effort must be made to support the system, and to maintain animal heat. If medicines are given at this stage, and are retained, they probably do harm. But while keeping up animal heat the freest ventilation must be secured. Raw meat soups may be *offered*, but should not be pressed on the patient, and only tea-spoons full should be given. This is often retained when everything else is vomited. The sudden cessation of vomiting when collapse is present, is always an unfavourable sign. The pulse may not be perceptible, the surface may be cold, and the voice reduced to a whisper, but "if," says Murray, "the patient has strength to vomit the case is not hopeless." Similarly so long as urine is passed the case should not be regarded as hopeless, and with the hope of encouraging this secretion mustard poultices may be employed over the loins. Good attendance and good nursing are the essentials for cholera patients. In conclusion, we warn against the employment of the nostrums which will doubtless be advertised should cholera approach this country. Some years back a German in Calcutta advertised the cure of cholera by injections of infusion of quassia, and, doubtless, much valuable time was lost in trusting to this means. Still, more recently a writer in the *Lancet* proposed the use of an epispastic solution behind the ears, which was asserted to stimulate the pneumogastric. A gentleman at Buenos Ayres used a cautery behind the right ear, which was also reported in the *Lancet*. Elliotson, years ago, tried mesmerism. The inhalation of oxygen gas was tried in England in 1849, and brought forward as a new treatment at Marseilles in 1884. But as Dr. Blanc, of Bombay, observed, "So far as drugging is concerned, almost total non-interference is the best;" and he will best treat the malady who, guided by the symptoms in individual cases, so combats the tendency to death. And Moore observes: "Successful treatment of cholera depends much on the sanitary surroundings of the patient, on the care that is taken in nursing and supporting him, not interfering with him too much, but carefully watching and treating the symptoms as they arise."

Although the treatment of cholera is thus to a great extent uncertain, experience shows that the prevention of cholera may be achieved. On the expectation of cholera the following are the principal points: Great attention to the general sanitary condition; avoidance of over-crowding; attention to ventilation; examination of traps, sinks, and drains, to see that they are in good working order; attention to the water supply. It cannot be too often reiterated that the incidence of cholera is on the most unsanitary localities. Personal hygiene should not be neglected, but radical alterations in the way of living should not be suddenly made. And *fear* of the disease should be combated; for fear is undoubtedly a great predisposing agent.

We observe it has been stated that the outbreak of cholera in Spain is ascribed to the disturbance of some old cesspools, which were in existence during a former epidemic. This is not the first time cholera has been attributed to very similar causes. Other maladies also have been similarly originated by *theory*. An epidemic of small-pox at Quebec was supposed to be due to the opening of a small-pox cemetery many years old. This entails the belief that disease germs may lay dormant for an indefinite period. An argument used in favour of this is the fact of wheat taken from the pyramids germinating. But the wheat was not in the earth, and earth is one of the best of disinfectants, deodorants, and decomposing agents. We are of opinion that we must search for the cause of cholera above the earth, and not below it.

